# Recent Advances in Polymer Composites for Flexible Pressure Sensors

**DOI:** 10.3390/polym15092176

**Published:** 2023-05-03

**Authors:** Wen-Tao Guo, Xin-Gui Tang, Zhenhua Tang, Qi-Jun Sun

**Affiliations:** School of Physics and Optoelectronic Engineering, Guangdong University of Technology, Guangzhou 510006, China

**Keywords:** polymer composites, pressure sensors, wearable electronics, health monitoring, electronic skin

## Abstract

Pressure sensors show significant potential applications in health monitoring, bio-sensing, electronic skin, and tactile perception. Consequently, tremendous research interest has been devoted to the development of high-performance pressure sensors. In this paper, recent progress on the polymer composite-based flexible pressure sensor is reviewed. The parameters of pressure sensors, including sensitivity, linear response range, detection limit, response speed, and reliability, are first introduced. Secondly, representative types of pressure sensors and relevant working principles are introduced and discussed. After that, the applications in human physiology monitoring, health monitoring, artificial skin, and self-powered smart system are listed and discussed in detail. Finally, the remaining challenges and outlook of polymer composite-based flexible sensors are summarized at the end of this review paper. This work should have some impact on the development of high-performance flexible pressure sensors.

## 1. Introduction

As an electronic device for detecting and transmitting information, the pressure sensor has the ability to perceive pressure signals and transform them into corresponding electrical signals. This enables the sensor to collect, transmit, process, analyze, and display information. Tactile perception [1,2,3,4], medical monitoring [5,6,7,8], wearable electronic products [9,10,11,12,13], and human motion detection [14,15,16] have emerged as popular application themes in the area of pressure sensors, especially in the previous few years. The classical pressure sensor ground on Micro-Electro-Mechanical Systems (MEMS) mainly consists of metal, semiconductors, piezoelectric crystals, etc., which are usually rigid materials. The technique of utilizing these materials to fabricate pressure sensors is proven and can precisely achieve remarkable performance in terms of low measurement errors and mass fabrication [17,18,19,20,21]. However, their shortcomings include the large volume and limited deformation. Others have hindered their application in human motion detection, health monitoring, and other scenes. Therefore, developing flexible pressure sensors that can bend and deform without losing performance is vital for expanding their potential uses in wearable devices and robotic systems.

**Figure 1:**
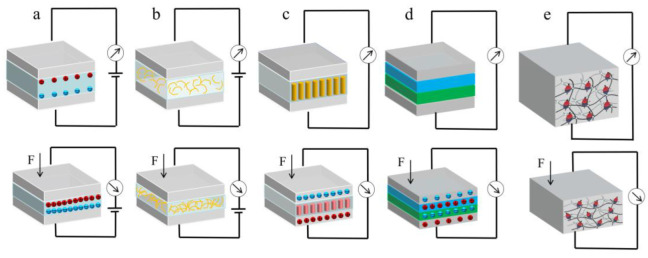
Pressure sensors with different working principles: (**a**) Capacitive; (**b**) Piezoresistive; (**c**) Piezoelectric; (**d**) Triboelectric; (**e**) Magnetoelastic.

Generally, based on different conduction mechanisms, pressure sensing devices can be basically classified into five types, including capacitive [9,11,22,23,24,25,26], piezoresistive [14,27,28,29,30], piezoelectric [15,31,32,33,34,35], triboelectric [36,37,38,39,40], and magnetoelastic [41,42,43] pressure sensors (Figure 1). In recent years, researchers have shown great research interest in capacitive and piezoresistive pressure sensors due to their straightforward device configuration and convenient signal processing. Meanwhile, piezoelectric, triboelectric, and magnetoelastic pressure sensors have the advantage of being self-powered, requiring no input external current or voltage to operate. While these sensors exhibit reliable dynamic pressure detection, achieving reliable static pressure detection remains challenging.

## 2. Parameters for Pressure Sensors

Pressure sensors with different operating principles have different sensitivity, stability, linear response range, detect limit, response time and output signals for different types of pressure (absolute, gauge, differential, or vacuum pressure). Therefore, when selecting flexible pressure sensors, appropriate pressure types and operating principles should be selected according to specific application scenarios and test objectives.

### 2.1. Pressure Sensitivity

The sensitivity (S) is expressed as the change in output signal relative to applied pressure, which is defined as S = (ΔE/E_0_)/ΔP. The initial electrical signal (such as current, capacitance, voltage, etc.) is denoted by E_0_, the relative variation of the corresponding signal is denoted by ΔE, and the change of the applied pressure is denoted by ΔP. Generally, sensitivity can be enhanced by decreasing the initial electrical signal E_0_. The relative variation in voltage output (current, capacitance) on the basis of external pressure is usually indicated, with the slope of the curve representing sensitivity. 

### 2.2. Linear Response Range

The linear response range refers to the range where the output is proportional to the input. In order to ensure a certain level of measurement accuracy, it is highly desirable for the relationship between the external stimuli input and relative electrical signal outputs to be linear. Theoretically, flexible pressure sensors have ideal linear models over a wide range of sensing, but in practice, the linear range is often only a part of the full range.

### 2.3. Detection Limit and Response Speed

The detection limit and response speed are crucial parameters that determine the effectiveness of a sensor. The detection limit refers to the minimum signal or the corresponding physical quantity that the sensor can measure with a certain precision or repeatability and is used to evaluate whether it is suitable for a given application. The response speed is how fast a sensor reacts to pressure changes. Real-time detection of stimuli signals such as heartbeat pulse, subtle human movements, and gas concentration necessitates a high response speed. In addition, when analyzing the parameter of response time, appropriate research methods should be selected according to different application scenarios and test purposes. If you only observe the response of a slow indicator, you do not need to change the pressure frequently. However, if you study the response of a precision tester, you have to consider the speed and accuracy of the testing equipment itself. Therefore, appropriate research methods must be considered when selecting sensors for these applications.

### 2.4. Reliability

The reliability of a pressure sensor refers to the ability of its components and equipment to maintain the same function and performance within a specified period of time. The higher the reliability of the sensor, the more stable and accurate it can measure pressure, and it can maintain its performance over a long period of use. In the application areas where the demand for sustained stability is not as high, as long as it is within the margin of error. In other high-reliability demanding applications, such as automotive, industrial automation, and aerospace, the reliability of pressure sensors is crucial because any failure can lead to loss or safety issues.

## 3. Working Principles and Examples of Polymer Composite-Based Flexible Pressure Sensors 

### 3.1. Capacitive

**Figure 2:**
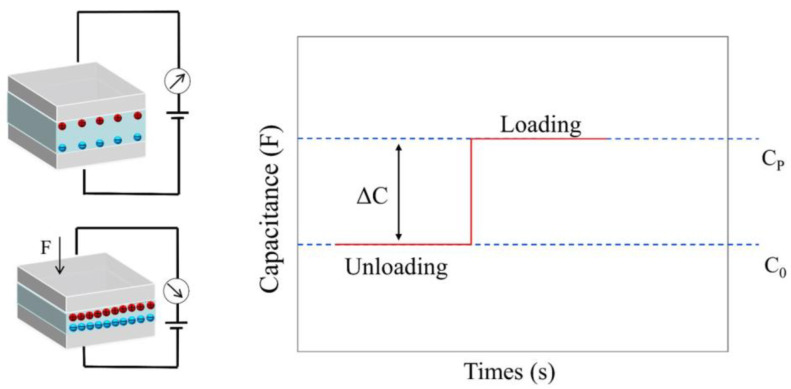
Working principle of capacitive pressure sensors.

Capacitive pressure sensors generally comprised up and down electrodes, a dielectric layer film, and a substrate. In comparison to other types of pressure sensors, capacitive pressure sensors consume less power, respond faster, and have less signal drift. The working mechanism of the capacitive can be demonstrated in Figure 2. External pressure changes the surface of contact from the dielectric materials to the electrodes and interelectrode distance, resulting in changes in capacitance. 

Neglecting the edge effect, we can use a simple governing Equation (1) to give the capacitance of a parallel plate capacitor:

(1) $$C={\varepsilon_{0}\varepsilon }_{r}A/d$$

The free-space permittivity and the relative permittivity of the dielectric material are denoted by ε_0_ and ε_r_, respectively. A is the area of the contact surface between dielectric materials and electrodes, and d is the interelectrode distance. When no external pressure is received, the sensor has little original capacitance ($C_{0}$) as a result of the interelectrode distance being relatively far. Under external pressure, the dielectric film deforms, and the capacitance increases to $C_{p}$. The sensitivity of the capacitive sensor can be calculated as the Equation (2):

(2) $$S=(\Delta C/C_{0})/\Delta P$$

The sensitivity and original capacitance are denoted by S and $C_{0}$, respectively. The variation in capacitance and applied pressure are expressed by ΔC and ΔP. For the purpose of enhancing the capability of the device, micropatterned structures can be generated in the dielectric layer by using templates or conventional lithography techniques. The most frequent shapes for microscale designs are pyramids [22,44,45,46], pillars [47,48,49], and porous structures [24,50,51,52].

**Figure 3:**
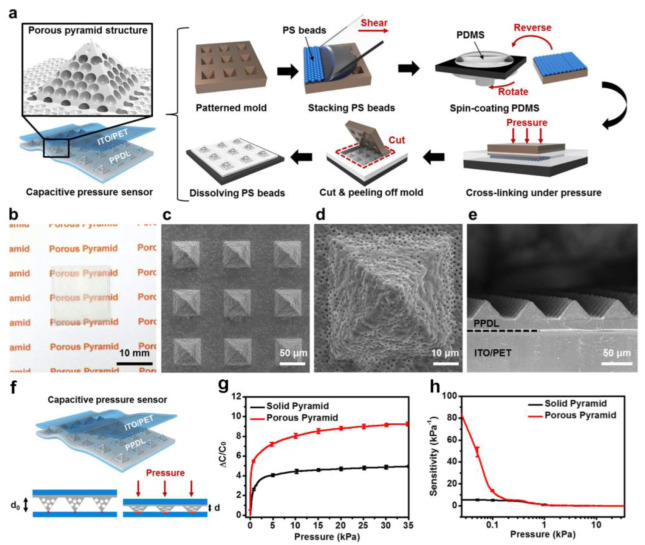
(**a**) Fabrication process of the PPDL pressure sensor. (**b**) An optical photograph of the PPDL. (**c**,**d**) The top view and (**e**) sectional view of an SEM photograph of PPDL. (**f**) The mechanism of PPDL pressure sensor. (**g**,**h**) The contrast of the capacitance (**g**) and sensitivity (**h**) against the pressure of the PPDL and SPDL pressure sensor. Reprinted/adapted with permission from Ref. [23]. Copyright 2019, American Chemical Society.

For example, according to the research of Yang et al., a capacitive sensor with a porous pyramid microstructure was much more sensitive than one with single micro-pyramids [23]. They achieved a sensitivity of 44.5 kPa ^− 1^ for a pressure range under 100 Pa. Figure 3a demonstrates the fabrication process of the porous pyramid dielectric layer (PPDL). First, prepare a pyramid microstructure- etched silicon mold and fill it with polystyrene (PS) beads by blade coating. A flexible PET/ITO (polyethylene terephthalate/indium tin oxide) substrate pre-cured with polydimethylsiloxane (PDMS) is then covered with the silicon mold that has beads coated on it. Under the action of pressure and heat, the PDMS infiltrated the cracks of the beads and their solidified products from a solidified state. Next, the mold is torn off from the substrate, leaving PDMS pyramids on the substrate. Finally, the beads were melted away by toluene, and then a piece of PET/ITO film was covered on the porous pyramids to finish the fabrication. Figure 3b renders an optical photograph of the PPDL, which is characterized by its high transparency. Figure 3c–e presents the scanning electron microscopy (SEM) photographs of the porous pyramids. As pressure is exerted, the pyramids become thinner, leading to a rise in capacitance, which is shown in Figure 3f. For comparison, a contrastive sensor based on a solid pyramid dielectric layer (SPDL) was also fabricated. Figure 3g, h exhibits the relative capacitance changes ($\Delta C/C_{0}$) and sensitivity curves of PPDL and SPDL with applied pressure, respectively. 

In summary, the PPDL exhibited significantly higher sensitivity for capacitive sensors than the SPDL. The PPDL structure allowed for more efficient deformation under pressure, leading to a greater change in capacitance. Therefore, micropatterned structures, such as the porous pyramid microstructure, offer a promising approach to raising the capability of capacitive sensors. Nevertheless, some limitations exist, such as it is only linear sensing in a limited pressure range. In addition, because the capacitance value is difficult to measure or process directly, a converter is required to convert the capacitance to the corresponding voltage signal.

**Figure 4:**
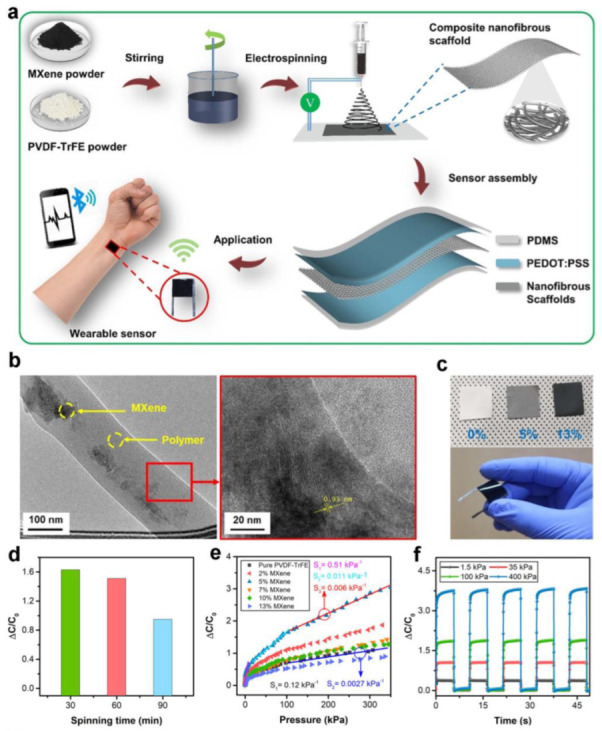
**(a**) Fabrication process of CNS-based sensor. (**b**) The TEM photograph of the CNS. The inset demonstrates sharp TEM. (**c**) Images of the sensors doped with diverse concentrations of MXene. (**d**) The effect of different electrospinning times on the performance of the sensor. (**e**) The difference in ΔC/C_0_ of the sensors with varying MXene concentrations in terms of weight percentage. (**f**) The variation of capacitance of the sensors with varying MXene concentrations under diverse external pressure. Reprinted/adapted with permission from Ref. [9]. Copyright 2020, American Chemical Society.

To solve the problem of small linear sensing range, a pressure sensor based on MXene composite nanofibrous scaffolds (CNS) was developed by Sharma et al. [9], which is illustrated in Figure 4a. The production process involved blending a 2D metal carbide, MXene (Ti_3_C_2_T_x_), with a polyvinylidene fluoride–trifluoroethylene (PVDF-TrFE) polymer using an electrospinning technique to prime the CNSs as a dielectric material. Finally, the resulting CNS was sandwiched between poly (3,4-ethylene dioxythiophene) polystyrene sulfonate (PEDOT:PSS)/PDMS films to complete the pressure sensor. Figure 4b presents nanofiber captured by transmission electron microscopy (TEM). The insets exhibit sharp TEM images of CNS. Figure 4c displays an optical photograph of the CNS under different doping concentrations of MXene. To explore how the thickness influences the sensor’s performance, the study in Figure 4d changed only the electrospinning time and kept other parameters constant. In total, three CNS were prepared with different spinning times (30, 60, and 90 min), and ΔC/C_0_ was measured under constant pressure. It can be analyzed that because the thinnest CNS has a large porous structure, the relative change of its capacitance is higher than the thickest one. Figure 4e exhibits the capacitance change under compression of 0.4 mm for different MXene concentrations. In the low-pressure stage, the sensor with 5% MXene showed an optimum sensitivity of 0.51 kPa^−1^, while the pristine PVDF-TrFE-based one had a sensitivity of only 0.12 kPa ^−1^. This difference in sensitivity is due to the reduction in compression modulus of the CNS with increasing filler concentration and the increase in relative dielectric permittivity. The lower compression modulus allows for greater deformation even at low stress, which increases sensitivity. Meanwhile, the higher relative permittivity leads to a greater change in capacitance, which also increases sensitivity. The capacitive response of samples under repeated compression/release was examined at a compression displacement of 0.3 mm, as shown in Figure 4f. The sensor of 5% MXene displays the highest ΔC/C_0_ compared to the other concentrations, which can be due to its higher sensitivity in both high and low-pressure ranges. In short, this sensor has the potential to monitor physiological signals and may work as a human–machine interface device for next-generation technology.

### 3.2. Piezoresistive

Piezoresistive pressure sensors generally consist of substrates and conductive materials which convert pressure changes into resistance or current change. Traditional piezoresistive pressure sensors use rigid substrates such as silicon and germanium, which have high piezoresistive coefficients but poor flexibility and stretchability. Substrates such as PDMS and PET are usually adopted in order to obtain good flexibility and stretchability. In addition to conducting electricity, conductive materials need to be sensitive to pressure changes. The principle of transforming external pressure into corresponding resistance variation is the foundation of piezoresistive pressure sensors, which can be regulated by the Equation (3):

(3) R=ρL/A

In this equation, ρ is the material’s specific resistance, L is the length, A is the area of the contact surface, and R is the resistance of the contact surface.

**Figure 5:**
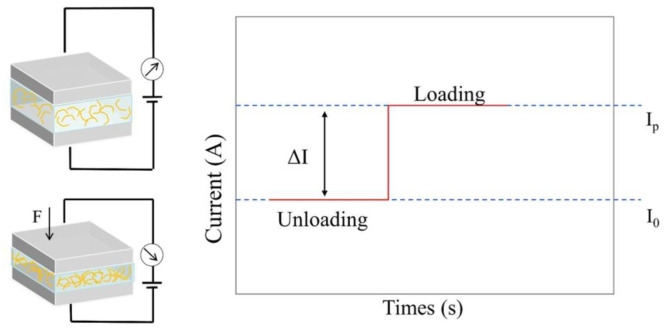
Working principle of piezoresistive pressure sensors.

As shown in Figure 5, under no-pressure conditions, the polymer nanocomposite film represents high resistance characteristics, and the sensor can only generate weak current ($I_{0}$). External pressure causes mechanical stresses of compression or tension on the piezoresistive pressure sensor, which results in an increase in the output current to $I_{P}$. Therefore, the piezoresistive pressure sensor, via the variation in resistance or output current, reflects the variation of external pressure. Similar to that of capacitive sensors, the sensitivity of a piezoresistive one is calculated by Equation (4)

(4) $$S=(\Delta I/I_{0})/\Delta P$$

The sensitivity S of the sensor is the ratio corresponding to the variation in current from the original value $I_{0}$ (the current without pressure) to ΔI under the condition of the variation in applied pressure ΔP, and also the ratio corresponding to ΔP.

**Figure 6:**
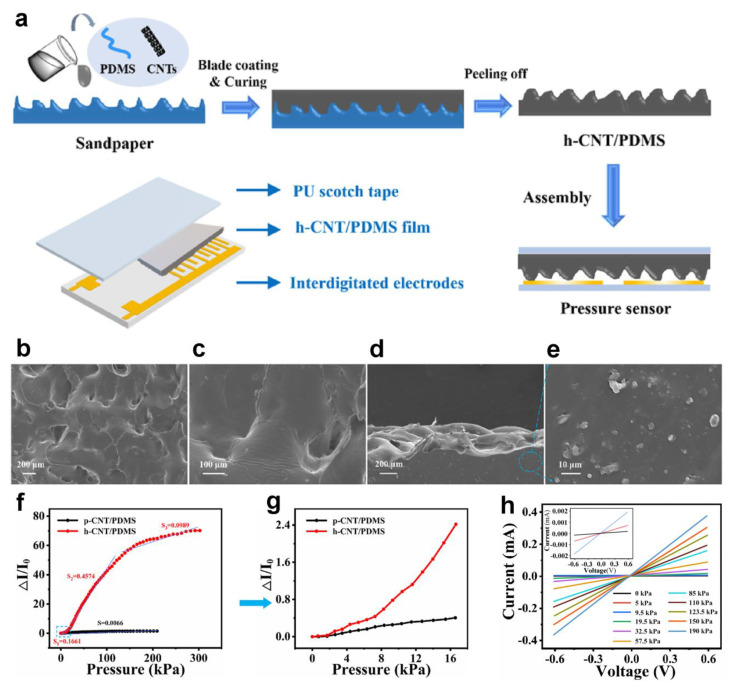
(**a**) The fabrication process of h-CNT/PDMS-based sensor. (**b**–**e**) SEM images of h-CNT/PDMS film with different magnifications. (**f**,**g**) Relative current variation of h- and p-CNT/PDMS-based pressure sensors under external pressure, (**g**) enlarged view in dotted box. (**h**) The current-voltage curve of the sensor under diverse pressures. Reprinted/adapted with permission from Ref. [58]. Copyright 2021, Elsevier.

Over the last several decades, various polymer nanocomposites have been utilized as pressure-responsive layers for piezoresistive pressure sensors, including carbon nanotube (CNT) [53,54], carbon black (CB) [55,56], and carbonyl iron powder (CIP) [57]. For instance, Zhao et al. developed a sensor based on hierarchically structured CNT/PDMS film (h-CNT/PDMS) by blade coating techniques adopting sandpaper as the substrate [58]. For comparison, the planar structured CNT/PDMS film (p-CNT/PDMS) adopting glass substrate with similar conditions was fabricated. Then, the microstructure film was assembled with electrodes and polyurethane (PU) tape to acquire the proposed sensor, as depicted in Figure 6a. The SEM photographs of microstructure film with different magnifications are displayed in Figure 6b–e. Obviously, plentiful hierarchical bulging structures with varying heights show on the surface. Figure 6f, g describes the relationship between $\Delta I/I_{0}$ and external pressure of hierarchical and planar structure sensors. Due to the microstructure of the hierarchical film, the contact area between the microstructure film and the interdigitated electrode varies remarkably when it is under pressure, compared with the plane film. Benefited from the special hierarchical structure, the hierarchical structure sensor shows upper sensitivity than the planar one. Figure 6h describes the current and voltage curves of the sensor under different pressures, which shows ohm linear characteristics. According to the authors, the sensor has well reliability (1500 cycles) and fast response time (100 ms), demonstrating its potential for applications.

**Figure 7:**
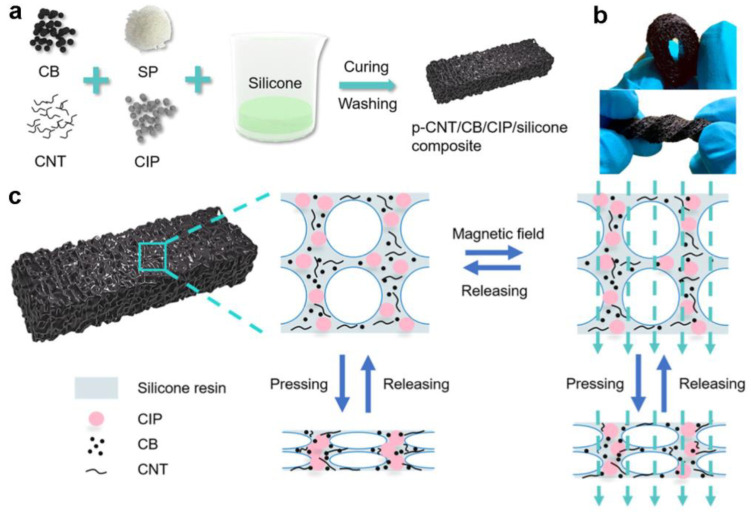
(**a**) Fabrication process of the composite. (**b**) Photograph of the composite when bent and twisted. (**c**) Working mechanism of the proposed sensor. Reprinted/adapted with permission from Ref. [16]. Copyright 2023, American Chemical Society.

Huang et al. developed a flexible pressure sensor with a wonderful linear response (R^2^ = 0.999) which was based on a flexible and porous CNT, CB, CIP, and silicone composite [16]. Figure 7a describes the fabrication of the p-CNT/CB/CIP/silicone composite. In brief, the flexible porous composite pressure sensor was developed by simple blending, curing, and washing methods. The composite sensor is prepared by introducing sugar particles (SPs) that are later removed to create a porous structure (Figure 7b). Figure 7c illustrates the working principle of the proposed sensor. The existence of a porous structure causes the composite film to have a smaller compressive modulus under a low-pressure range, which enhances the ability to detect the smaller pressure of the proposed sensor. In summary, piezoresistive pressure sensors have a preponderance of their uncomplicated design, good linear response, high sensitivity, and low price, but they also have some shortcomings such as temperature sensitivity, drift, and fatigue.

### 3.3. Piezoelectric

**Figure 8:**
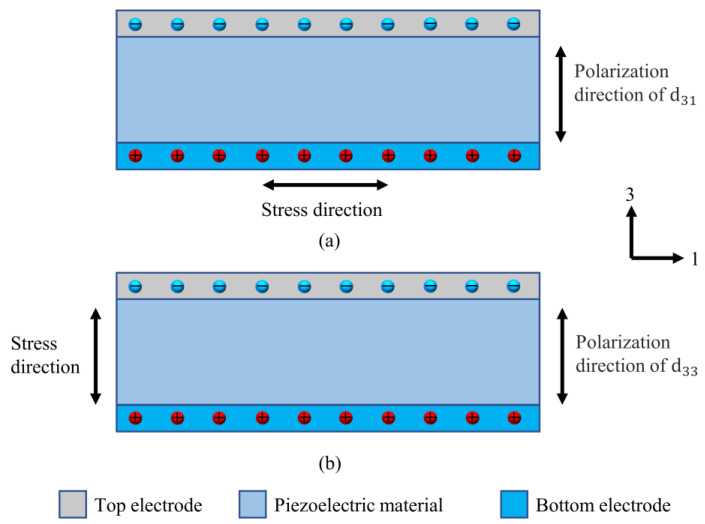
The two working modes of piezoelectric energy collector. (**a**) $d_{31}$ working mode, (**b**) $d_{33}$ working mode.

Piezoelectric nanogenerators (PENGs) have been widely studied for generating electricity from minor mechanical forces and acting as self-powered devices since the PENG based on ZnO nanowire arrays was developed in 2006 [59]. In brief, the induction mechanism of PENGs is derived from the piezoelectric effect of piezoelectric materials that uses the piezoelectric polarized charge and the electric field, which varies with time to generate an electric current in the circuit. The mechanism of piezoelectric sensors is expressed by Equation (5):

(5) $$Q=d_{ij}F$$

where Q is the charge generated, $d_{ij}$ (i = 1,2,3, j = 1,2,3, i on behalf of the direction of crystal polarization and j on behalf of the direction of force) is the piezoelectric coefficient, and F is the external force, respectively. Generally, $d_{31}$ mode and $d_{33}$ mode are two common working modes of piezoelectric energy collectors. Figure 8 describes the schematic diagram of these two working modes. When the piezoelectric sensor works in $d_{31}$ mode, both the stress and the mechanical deformation of the piezoelectric material are along the direction 1. Meanwhile, the polarization of dielectric materials and the electric field produced are in direction 3. When the piezoelectric sensor works in $d_{33}$ mode, the stress, the mechanical deformation of the dielectric material, the polarization of dielectric materials and the electric field produced are all in three directions. The most important parameter describing the piezoelectric properties of dielectric materials is the piezoelectric coefficients $d_{ij}$, which is reflected in the ability of dielectric materials to generate electricity in turn or from minor mechanical forces. In general, a larger $d_{ij}$ indicates better piezoelectric properties of dielectric materials. The piezoelectric materials that are normally used are polymers and inorganic materials. These include lead zirconate titanate (PZT) [60,61,62,63,64], ZnO [65,66,67,68], polyvinylidene fluoride (PVDF) [69,70,71,72,73,74], GaN [32,75], Barium Titanate Oxide (BTO) [31,35,76], and others. PENGs have the characteristics of uncomplicated design and lasting durability, making them beneficial to power micro/nano systems and low-power consumption devices. 

**Figure 9:**
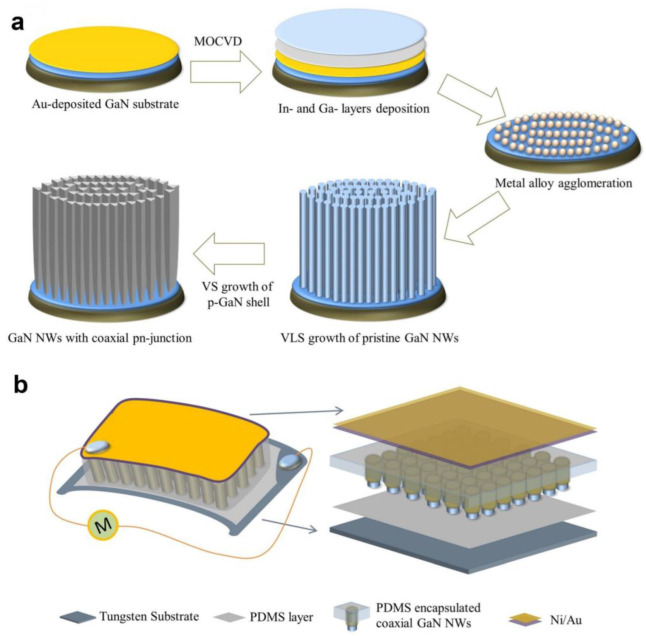
(**a**) Illustration of the synthesis of pristine and coaxial GaN NWs. (**b**) Design of the proposed sensor. Reprinted/adapted with permission from Ref. [32]. Copyright 2021, Elsevier.

Waseem et al. developed a flexible piezoelectric pressure sensor (PEPS) based on p-n junction coaxial GaN nanowires (NWs) [32]. Figure 9a describes the growth process of GaN NWs using MOCVD. Pristine GaN NWs were grown on a GaN thin film covered with an Au film using an electron beam evaporator, while the p-GaN shell was produced by introducing bis(cyclopentadienyl)magnesium (Cp_2_Mg) for p-type doping. By adjusting the flow rates of Cp_2_Mg, coaxial GaN NWs with heavy, medium, or low doping levels were fabricated. Figure 9b illustrates the typical design of the proposed sensor. According to researchers, unlike pressure sensors based on semiconductor piezoelectric materials, the proposed PEPS can measure both dynamic and static pressure due to a greatly suppressed internal screening. The PEPS spends 19150 s for voltage decaying, which is the largest among semiconductor-based PEPSs, indicating that the sensor has good stability and reliability. It also has a linear response of R^2^ = 0.992, high sensitivity of 14.25 V·kPa^−1^, and a rapid response time of 55 ms.

**Figure 10:**
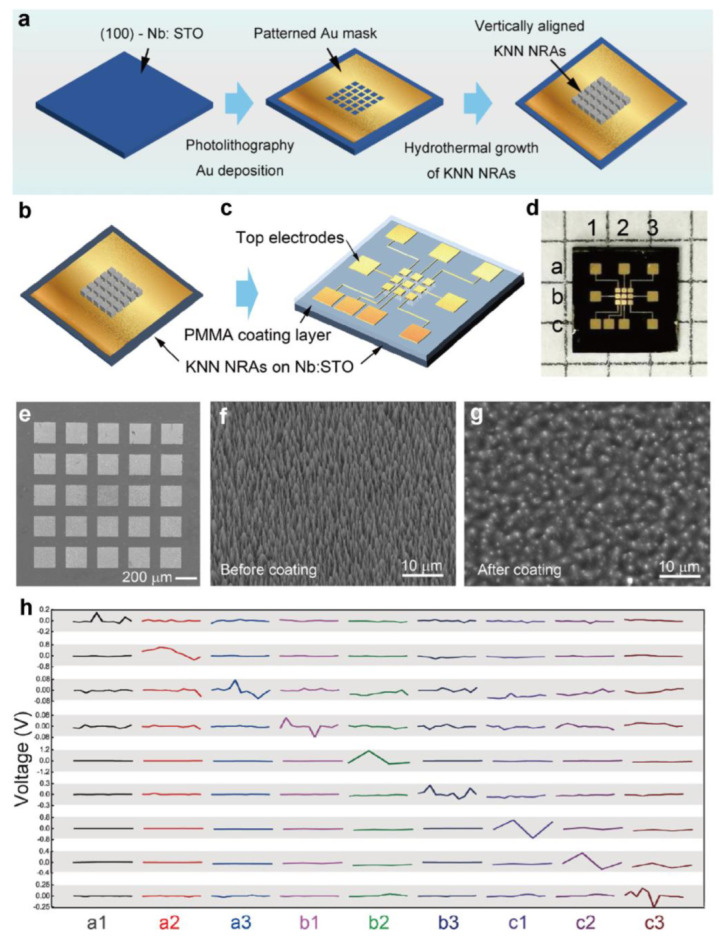
(**a**) Fabrication method of the dot matrix of KNN NRAs. (**b**,**c**) Fabrication process of the micro-sensor units. (**d**) Optical image of the micro-sensor units. (**e**–**g**) SEM images of the 5 × 5 dot matrix of the KNN NRAs, the KNN nanorods before and after PMMA coating. (**h**) Output signal while a single dot is pressed sequentially. Reprinted/adapted with permission from Ref. [77]. Copyright 2023, Springer Nature.

Jiang et al. developed a pressure sensor matrix capable of two-dimensional pressure mapping by utilizing patterned nanorods arrays (NRAs) of piezoelectric potassium sodium niobate ((K,Na)NbO_3_ (KNN)) [77]. The process of synthesizing patterned KNN NRAs is illustrated in Figure 10a. To obtain the aligned growth of the KNN nanorods, researchers used the Nb:STO single crystal as the template. After photolithography and magnetron sputtering, the substrate is covered with a patterned gold mask that serves as a barrier layer, enabling the nanorods to grow in the desired area. A micro-scale dot pattern consisting of vertically grown KNN nanorods was prepared by hydrothermal method. After that, the researchers constructed the microsensor array by depositing a 3 × 3 dot matrix top electrode on the PMMA-coated NRAs, as depicted in Figure 10b–e. Except where the top of the nanorods connects to the electrode, the rest of the nanorods are coated with PMMA to prevent electrode leakage and breakage (Figure 10f,g). Each sensor had an effective area of 200 μm × 200 μm with a gap of approximately 100 μm between adjacent units. The sensor matrix size was determined using finite-element-simulation to measure the distribution of pressure caused by a pen-point with a diameter of 0.25 mm. The fabrication technique was flexible, allowing for customization to meet different application needs. As shown in Figure 10h, tests were conducted on each sensor unit by using random forces through a 0.25 mm pen-point. The touched point emitted a clear voltage signal, while signals from other points were interference. Overall, the sensing matrix shows high sensitivity of 0.20V/N per sensor unit and a minimum detection limit of 20g. Due to the similarity of the KNN NRAs sensing units, the sensitivity of each NRA point is close, so the external pressure acting on the entire sensing matrix can be estimated by the output voltage with a resolution of 0.2 mm. This indicates its capability for application in electronic skin and tactile perception.

### 3.4. Triboelectric

Triboelectric effects and electrostatic induction are the basic principles that triboelectric pressure sensors operate on. For triboelectric generators (TENGs), there are normally two triboelectric layers that face each other. Under external force, the triboelectric layers contact each other and will generate opposite tribo-charges. In addition, two insulated electrodes allow the charge to be transferred between the two electrodes through an external circuit. The transfer charge from one electrode to another is defined as Q, and then the transfer charge of the two electrodes is Q and -Q. There are two parts consisting of the voltage drop across the two electrodes of a TENG. The polarized triboelectric charges produce the first part, which is expressed as $V_{oc}(x)$, the other part is from the already transferred charges Q [78]. If the construction of TENG is considered a typical capacitor, the transferred charge between the two electrodes is Q/C(x), where C represents the capacitance entre the two electrodes. By applying the additivity property of electric potential, we can obtain the fundamental equation of TENGs as Equation (6):

(6) $$V=-\frac{1}{C\left(x\right)}Q+V_{oc}(x)$$

**Figure 11:**
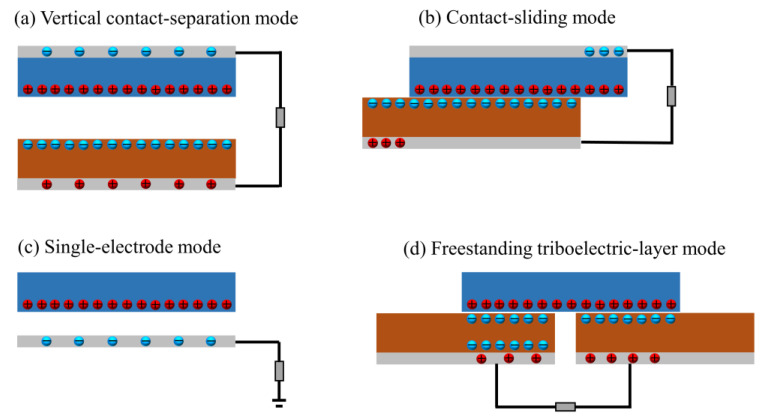
Four TENGs modes: (**a**) Vertical contact-separation mode. (**b**) Contact-sliding mode. (**c**) Single-electrode mode. (**d**) Freestanding triboelectric-layer mode.

Between the two electrodes of TENG, V is the total voltage, C is the capacitance, x is how far apart the two triboelectric layers are, Q is how much charge is transferred, and $V_{oc}$ is the voltage effect of the polarized triboelectric charges. Consequently, there are four distinct work modes for TENGs, as shown in Figure 11, including vertical contact separation mode, contact sliding mode, single electrode mode, and freestanding triboelectric layer mode [78]. Vertical contact separation mode has attracted extensive research due to its benefits of higher output performance, convenient fabricated process, and various material options, which can be readily integrated with any surface to harness the energy of contact and separation.

**Figure 12:**
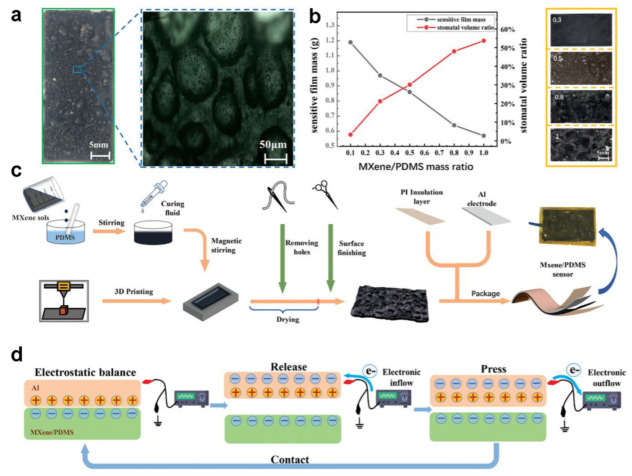
(**a**) Photograph of MXene/PDMS composite film and high-resolution zoom. (**b**) a plot of the porosity of the composite film against the mass ratio and images of composite films with different porosity. (**c**) The diagram for fabrication process. (**d**) The operating principle of the proposed sensor. Reprinted/adapted with permission from Ref. [51]. Copyright 2023, Wiley-VCH.

As an illustration, Wang et al. proposed an MXene/PDMS composite-based flexible triboelectric pressure sensor [51]. Figure 12a describes the prepared film and microscopic photographs of the Mxene/PDMS structure. As the blend is dried, the moisture content is removed, resulting in the formation of numerous small and irregularly shaped pores (with diameters ranging from 100 μm to 1 mm) throughout the film. In that work, five different film samples were fabricated using Mxene colloid and PDMS solution at varying mass ratios of 0.1, 0.3, 0.5, 0.8, and 1, with different masses and stomatal volumes. Figure 12b displays a plot of the porosity of the composite film against the mass ratio. As illustrated in it, when the mass ratio is 0.5, the film can maintain its morphological integrity and good mechanical properties while having satisfactory porosity. Figure 12c presents the diagram of the fabrication process. Aluminum foil is used as a positive triboelectric layer. A hydrothermal oxidation reaction was performed by immersing the electrodes in deionized water and heating them at 80 °C for 2 h, producing the emergence of irregular nanostructures on the Al foil surface. The final sensor was formed by combining the porous MXene/PDMS film with the Al foil and a Cu wire encapsulated with commercial Kapton film. Figure 12d describes the operating principle of the proposed sensor. The sensor produces electric pulses through contact-separation cycles, and positive and negative charge distributions are generated when subjected to pressure. The triboelectric charging effect occurs between the positive triboelectric layer (Al foil) and the composite film (Mxene/PDMS), resulting in electron transfer. The cyclic contact-separation action creates a voltage drop across the positive and the negative layer, generating electric pulses without external power.

**Figure 13:**
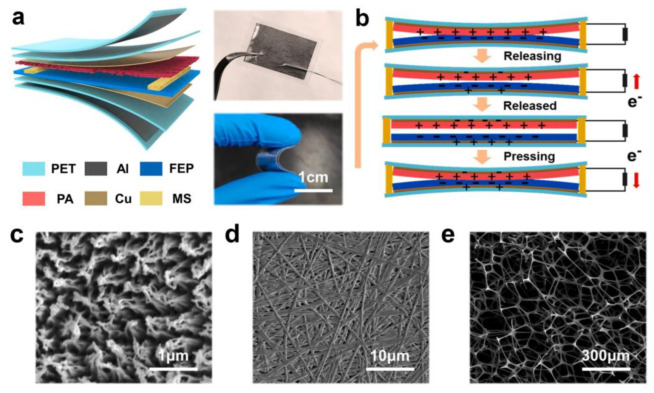
(**a**) Schematic structure diagram and the optical photographs of the sensor. (**b**) Working principle of the sensor. (**c**–**e**) SEM images of the FEP film, PA film, and MS spacers, respectively. Reprinted/adapted with permission from Ref. [79]. Copyright 2021, Elsevier.

Xu et al. developed a self-powered triboelectric pulse sensor for blood pressure monitoring [79]. Figure 13a exhibits the schematic structure diagram and the images of the constructed sensor. The TENG-based sensor device has been constructed using two triboelectric layers (Fluorinated ethylene propylene (FEP) and Polyamide (PA)), electrodes (Cu), spacers (melamine sponge (MS)), electrostatic shielding layers (Al). Finally, the entire device has been enclosed within encapsulating layers (PET). Figure 13b illustrates that the mechanism of the proposed sensor depends on the combination of triboelectric and electrostatic induction. Triboelectrification occurs primarily as a result of surface electrons being transferred from PA to FEP, which creates a potential difference between the two layers, creating an alternating current output. As shown in Figure 13c–e, FEP film, PA film, and MS spacers were imaged by SEM, respectively.

TENGs are known for their high output performance and simple fabricated processes. However, they rely on the contact separation motion of two dielectric materials to generate electric energy, which limits the type of mechanical energy that can be used. In contrast, PENGs can transfer charge efficiently and only depend on the deformation of the dielectric material to generate electric energy. While the voltage output performance of PENGs is typically lower than TENG. Therefore, developing a hybrid triboelectric/piezoelectric nanogenerator is a promising approach that can overcome the limitations of both TENG and PENG devices. As a matter of fact, piezoelectric and triboelectric materials can achieve both deformation and friction through rational structural design. To improve sensitivity, linear sensing range, and wearing comfort of wearable electronics, the idea of a hybrid sensor that combines piezoelectricity and triboelectric effects is proposed, which can work as PENG and TENG at the same time. The development of a hybrid piezoelectric–triboelectric compound sensor has drawn significant attention due to its potential for high-performance energy harvesting. Zhang et al. exploited an r-shaped hybrid piezoelectric–triboelectric sensor in 2013 [80], which laid the foundation for the further exploration of hybrid energy harvesting technology. In 2016, Wang et al. presented a flexible hybrid triboelectric–piezoelectric sensor based on PDMS/MWCNT [81], which improved the output capability by employing PDMS patterned films containing multiwall carbon nanotubes (MWCNT) as a triboelectric layer. To further raise the flexibility and permeability of the hybrid TENG, Guo et al. introduced an all-fiber hybrid TENG enhanced by piezoelectric materials in 2018 [82], which offers the same advantages as regular fabrics. Together, these examples demonstrate the potential of hybrid piezoelectric–triboelectric nanogenerators in energy harvesting and the continuous innovation and optimization of this technology. Therefore, a hybrid piezoelectric–triboelectric nanogenerator with multifunctions of simultaneous power generation and sensing is necessary.

**Figure 14:**
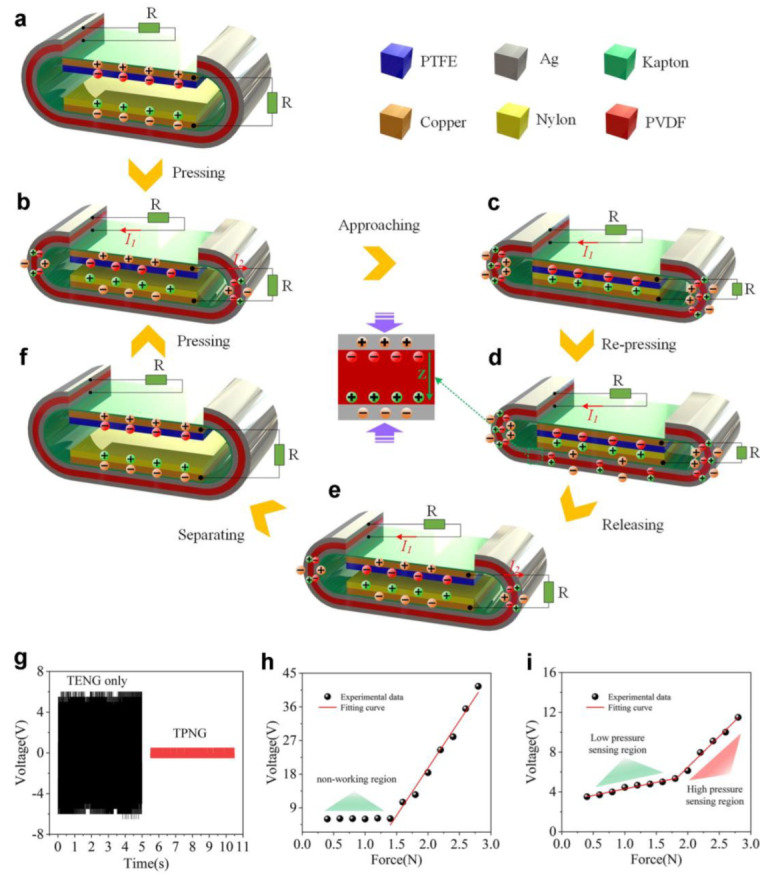
(**a**–**f**) Structure and working principle of the TPNG. (**g**) The noise voltage of the triboelectric part and TPNG. (**h**) The output voltage of the triboelectric part. (**i**) The output voltage of the TPNG. Repinted/adapted with permission from Ref. [83]. Copyright 2022, Elsevier.

Song et al. developed a self-power structure hybrid triboelectric–piezoelectric nanogenerator (TPNG) [83], which comprises a triboelectric part (polytetrafluoroethylene (PTFE) and nylon materials) and a piezoelectric part (polyvinylidene fluoride (PVDF)). Figure 14a–f illustrates the structure and mechanism of the TPNG. Negative and positive charges are created by frictional contact between the PTFE and Nylon films. The piezoelectric part creates charges and current in circuit 1 when pressed. The triboelectric part also creates current in circuit 2 by electrostatic induction. Only circuit 1 has a current when the films come into contact. Circuit 1 continuously has a current when more pressure is applied. The TPNG springs back and reverses the current in both circuits when the pressure is removed. Both circuits have alternating currents under repeated pressure.

With the above principle, the researchers developed a self-powered pressure sensor based on TPNG. Compared to the triboelectric part by itself, TPNG can reduce noise, as depicted in Figure 14g. The triboelectric part of the sensor could not sense force in the range of 0.4 to 1.4 N due to the supporting structure, as depicted in Figure 14h. However, when the pressure exceeded 1.4 N, the TPNG could accurately sense the pressure change, as demonstrated in Figure 14i. In the low-pressure range (0.4–1.6 N), the sensitivity and linearity of the proposed sensor were 1.30298 V/N and 0.97873. In the high-pressure range (1.6–2.8 N), the sensitivity and linearity were 6.2249 V/N and 0.98868. Therefore, the proposed sensor using TPNG offers a wider sensing range.

Overall, the developments of hybrid piezoelectric–triboelectric nanogenerators have garnered substantial recognition due to their potential for high-performance energy harvesting and sensing applications. This hybrid structure unites the merits of both TENG and PENG, enabling the simultaneous generation of triboelectric and piezoelectric signals. Consequently, the hybrid piezoelectric–triboelectric nanogenerator has shown great potential in overcoming the limitations of traditional devices and achieving multifunctional power generation and sensing.

### 3.5. Magnetoelastic

**Figure 15:**
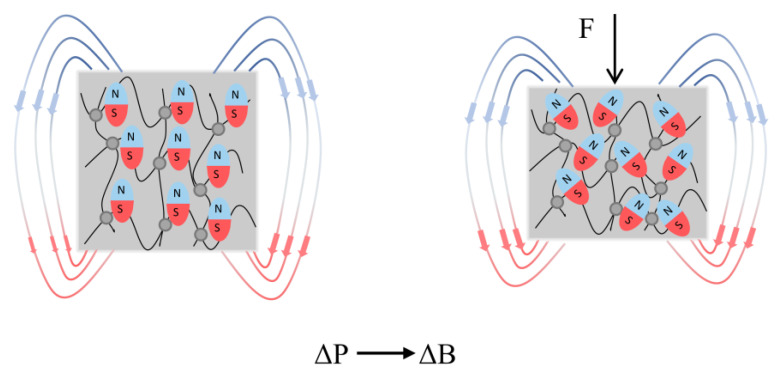
The principle of wavy chain structure in magnetoelastic sensor.

Since Chen et al. reported the giant magnetoelastic effect in soft matter two years ago, the magnetoelastic sensor has been drawing great focus to their applications in flexible and stretchable electronic devices due to its water impermeability, brilliant biocompatibility, low cost, and long life [42]. Under an applied magnetic field, metal alloys like Tb_x_Dy_1−x_Fe_2_ and Ga_x_Fe_1−x_ exhibit magnetoelastic effects [41]. Nevertheless, its applications in the field of biological electronics have been neglected because of the little change of magnetization under mechanical stress conditions and structure intricacy caused by the applied magnetic field. The magnetoelastic effect refers to the phenomenon that the magnetic properties of a material change when subjected to mechanical stress such as force or torque. The principle of the magnetoelastic effect was examined by building a wave chain model (Figure 15). The magnetoelastic effect of traditional metal alloys is due to the magnetic anisotropy caused by the rearrangement of the magnetic domain and mechanical stress, while the magnetoelastic effect occurs in the proposed soft matter results from the variation of micromagnet chain structure with mechanical strain. In the absence of applied pressure, the magnetic particles within the magnetoelastic layer are single magnetic dipoles arranged in wavy chains, which remain steady after magnetization. When compressed, the micromagnet chain structure changes, altering the dipole–dipole interaction within the chain and reducing the surface magnetic-flux density due to the demagnetizing field. When uniaxial stress is relieved, the recovery of the micromagnet wavy chain reinstates the magnetic-flux density to its initial state. The model of a wavy chain can be used to express how vertical magnetic field H_⊥_ and main stretch λ are related [42], which can be expressed as Equation (7)

(7) $$H_{⊥}\approx -\frac{1}{\left(2a+1\right)\lambda^{1.5k}}M+\frac{r^{3}M}{3\lambda^{3}h^{3}}\left(0.3006-f\left(\frac{b}{h\lambda^{1.5}}\right)\right)$$

The variables in the equation include the radius of the magnetic particle (r), the aspect ratio of the wavy chain structure (a), the magnetization of the micromagnets (M), the influence constant (k), the aclinic and perpendicular distances between neighboring micromagnets (b and h), and the dipole alignment factor (0.3006-f(x)) which represents the contribution of all other dipoles to the vertical magnetic field of a single dipole. The magnetic-flux density of a flexible magnetoelastic film can be changed by external stress, which, due to the stress, can induce magnetic particle interaction and magnetic dipole interaction within the chain. When slight pressures are applied to the soft magnetoelastic film, giant magnetoelasticity results in a partial change in magnetic-flux density. According to the laws of electromagnetic induction (Equation (8)), magnetic energy can be additionally transformed into electrical energy.

(8) $$\varepsilon =-N\times \frac{d\Phi }{dt}$$

in which the magnitude of the voltage ε is determined by the number of turns N in the coil, the magnetic flux Φ and the time t. Therefore, a magnetoelastic generator has been created that utilizes flexible magnetoelastic films to transform mechanical energy into magnetic energy, which is then further transformed into electrical energy through the use of textile coils.

**Figure 16:**
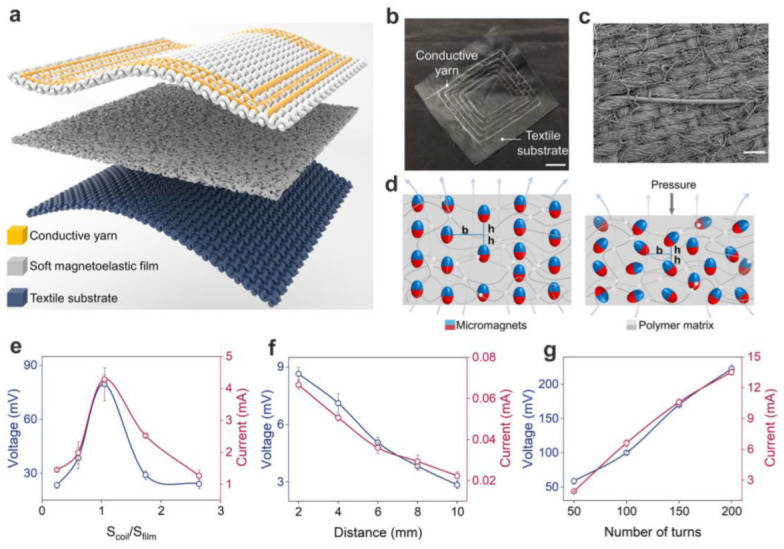
(**a**) Schematic of the structure of the textile MEG. (**b**) Optical image of the textile coil. (**c**) SEM image of the textile coil. (**d**) Wavy chain structure model of the magnetoelastic effect. (**e**–**g**) How the electric outputs of the textile MEGs vary with the size (**e**), distance (**f**), and number of turns (**g**) of the textile coil. Reprinted/adapted with permission from Ref. [43]. Copyright 2021, Elsevier.

According to the above principles, Chen et al. presented a type of textile magnetoelastic generator (MEG), which is shown in Figure 16a [43]. In this instance, a flexible magnetoelastic film was utilized for magneto–mechanical coupling in conjunction with a textile coil for electromagnetic reaction to create a textile MEG (Figure 16b). The textile coil was constructed by machine-sewing conductive yarns onto a textile substrate (Figure 16c). As depicted in Figure 16d, the wavy chain structure explains the magnetoelasticity in flexible film. If external pressure is not present, the micromagnets embedded in the soft magnetoelastic film form a stable wavy chain structure after being impulsively magnetized. However, when pressure is exerted on the flexible composite film, it can penetrate the polymer matrix and deliver a continuous source of energy for the micromagnetic particles to travel and migrate, which results in a decreased magnetic-flux density. Figure 16e–g describe the voltage and current outputs of the MEGs. To begin with, Figure 16e shows the relationship between the output and S_coil_ / S_film_. A smaller textile coil (S_coil_ / S_film_ < 1) is not capable of capturing the full range of magnetic-flux density changes in the compressed soft composite film, while a larger textile coil (S_coil_ / S_film_ > 1) contains opposing magnetic field lines that counteract each other. As a result, an optimal ratio for converting biomechanical energy to electrical energy is achieved when the size of the textile coil is approximately equal to that of the flexible composite film. Secondly, the distance between the two components was investigated, and the result shows that the output power of the textile coil is the highest when it is fitted closely to the flexible composite film (Figure 16f). Lastly, as exhibited in Figure 16g, the output has an obvious linear relationship with the number of turns of the textile coil, which is consistent with the law of electromagnetic induction (Equation 8). Therefore, due to the characteristics of hydrophobicity and self-power, the sensor based on MEG shows an excellent application prospect in motion detection and physiological signal detection.

In brief, due to the magnetic field that can travel through water, pressure sensors based on MEG have the potential for underwater health monitoring and disease diagnosis without encapsulation. However, to fully utilize these sensors for medical applications, it is significant to explore eco-friendly and biocompatible magnetoelastic materials.

## 4. Applications of the Polymer Composites for Pressure Sensors

### 4.1. Human Physiology Monitoring

**Figure 17:**
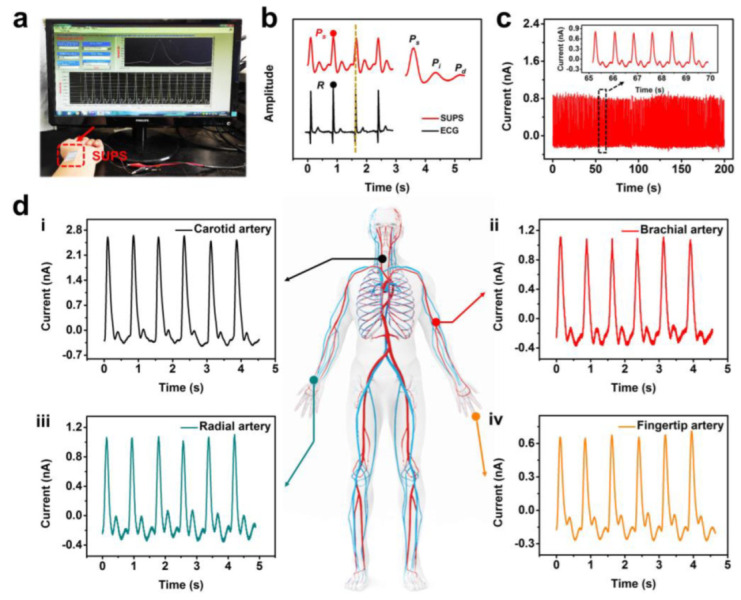
(**a**) The radial artery pulse waveforms monitored by the pulse sensor. (**b**) Comparison of pulse waves measured by electrocardiogram and pulse sensor. (**c**) Pulse waves from a male volunteer measured by pulse sensor. (**d**) Pulse waveforms monitored from diverse arteries (i) carotid, (ii) brachial, (iii) radial, and (iv) fingertip. Reprinted/adapted with permission from Ref. [79]. Copyright 2021, Elsevier.

In the previous few years, wearable electronics based on flexible pressure sensors have gained increasing popularity for monitoring human physiology due to their noninvasiveness, comfort, and accuracy. These devices have been employed for a diversity of applications, such as monitoring blood pressure [84,85,86,87], heartbeat [88,89,90], pulse [79,91,92,93], and other vital signs. In particular, pressure sensors attached to the skin surface have shown great potential for noninvasive detection of the pulse waveform. This capability has led to the development of the self-powered ultrasensitive pulse sensor (SUPS) [79] by Xu et al., which is a pulse detection network comprising pressure sensors distributed at different pulse sites that can simultaneously monitor multiple cardiovascular indicators. Figure 17a shows the pulse waveform signals monitored by the sensor attached to the radial artery. Figure 17b illustrates that the Systolic peak (P_s_) in the pulse waveform aligns accurately with the R waveform peak in the electrocardiograph signal, which illustrates the reliability of the proposed sensor for pulse detecting. The extended image of Figure 17b can clearly recognize the specific peaks Ps, Pi (reflected pulse wave) and Pd (left atrial ejection) in the single pulse waveform. Figure 17c depicts an image of the pulse sensor detecting the pulse of a male volunteer. Figure 17d shows the pulse wave signals detected by carotid, brachial, radial, and fingertip arteries. The different pulse waves measured at different parts of the body in Figure 17d are due to the fact that the pulse propagation speed is determined by factors including the muscle tissue structure, the elasticity of the vascular wall, and the peripheral resistance. The versatility and efficiency of the proposed pulse sensor—which has a high sensitivity of 10.29 nA·kPa^−1^, a response time of 30 ms, reliability of 20,000 cycles, and a detection limit of 5 mg—is expected to provide patients with a better monitoring experience, and facilitate more timely communication and treatment advice between doctors and patients.

**Figure 18:**
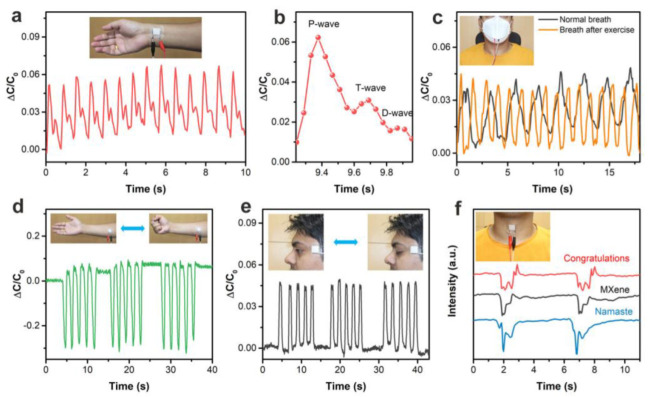
Application of the pressure sensor in detecting human physiological signals. (**a**) Image of detecting the artery pulse waveform. (**b**) Enlarged image of the pulse waveform signal. (**c**) Image of detecting human breathing patterns before and after exercise. (**d**) Image of monitoring muscle movement of the arm. (**e**) Image of detecting ocular muscle vibration. (**f**) Ability of the sensor to recognize words that sound different. Reprinted/adapted with permission from Ref. [9]. Copyright 2020, American Chemical Society.

Similarly, the CNS-based pressure sensor developed by Sharma et al. [9] successfully monitored human physiological signals. The capability of this sensor to accurately and precisely recognize the radial pulse, as illustrated in Figure 18a, demonstrates its potential as a medical diagnostic tool for monitoring heart rate and rhythm and identifying cardiovascular diseases. Figure 18b provides an enlarged image of the pulse waveform, exhibiting the distinctive peaks D-wave, T-wave, and P-wave, which correspond to the diastolic, tidal, and percussion responses of a pulse waveform, respectively. Figure 18c demonstrates that the proposed sensor can accurately detect human breathing patterns before and after exercise. Figure 18d illustrates that the sensor can be utilized to monitor muscle movement and capacitance changes caused by the contraction of the abdominal muscles of the arm. Furthermore, Figure 18e demonstrates the proposed sensor’s remarkable capability to detect ocular muscle vibration. Figure 18f depicts how the sensor attached to the throat produces different signals depending on the sound of different words, such as “Congratulations”, “MXene”, and “Namaste”. This is a good indication of the promising application of this sensor in the area of speech recognition. The portability and ease of this device could improve patients’ diagnosis and treatment experience, making them more willing to undergo routine examinations and monitoring.

**Figure 19:**
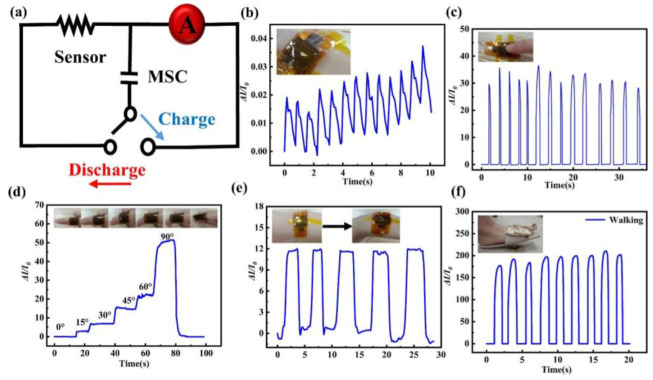
(**a**) Diagram of integrated sensing device. (**b**) Detect images of human pulse waveform signals. (**c**) Detect images of finger clicking. (**d**) Detected images of a finger bent at different angles. (**e**) Detect images of a wrist bent. (**f**) Detect images of walking. Reprinted/adapted with permission from Ref. [24]. Copyright 2023, Elsevier.

Wang et al. presented an integrated sensing system that can generate its own power and comprises a flexible pressure sensor and supercapacitor [24]. These devices are constructed using layered porous composites of Cu@Cu2O and graphitic carbon, which can be used to fabricate both pressure sensors and supercapacitors. The researchers integrated these two devices together to form a self-powered system. The resulting materials exhibit excellent flexibility, enabling their integration into a range of wearable electronics and flexible devices. As shown in Figure 19a switch, capacitor and sensor are connected in series to form a closed circuit. Human physiological signals were monitored by the self-powered sensing device. In Figure 19b, the flexible sensor and supercapacitor were taped to the human skin, which was able to detect real-time human pulse waveform signals. As depicted in Figure 19c, with a quick tap of the finger, the device responds to changes in pressure and outputs sharp electrical signals. The device provides high sensitivity and fast response time. In addition, the device was placed on the index finger to test the curved motion. The sensor generated stable current signals regardless of whether the finger was bent at fixed or varying angles, as shown in Figure 19d. The researchers also attached the proposed sensor to the volunteer’s wrist, which demonstrated increased sensor current with increasing wrist bending angle, as depicted in Figure 19e. As revealed in Figure 19f, the pressure sensor was able to detect the walking state of the participant, indicating magnificent performance in the wise pressure sensing range. These examples illustrate the immense potential for flexible pressure-sensing devices to transform the realm of human physiology detecting and open up possibilities for novel and innovative healthcare solutions.

### 4.2. Health Monitoring

The flexible pressure sensors, made from flexible materials and can be produced in various shapes, have characteristics such as lightness, thinness, flexibility, and bendability. Therefore, it has broad application prospects in medical monitoring. 

**Figure 20:**
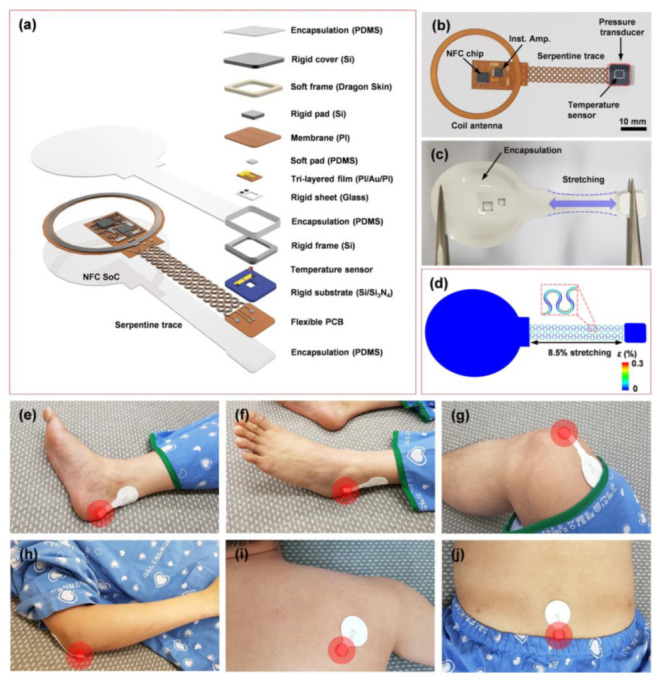
(**a**) Diagram of the sensing device. (**b**) Image of the device before packaging. (**c**,**d**) Images of a packaged device in a stretched configuration and its corresponding FEA results. (**e**–**j**) Images of devices attached to different positions of the body, including the heel, malleolus, knee, elbow, scapulae and sacrum. Reprinted/adapted with permission from Ref. [6]. Copyright 2021, Springer Nature.

Oh et al. developed a flexible, skin-compatible sensor system which can steadily monitor pressures and temperatures on critical skin interfaces [6]. The system uses a self-powered and wireless pressure-sensing device that can measure the pressure at diverse locations on the human body. The system is based on the membrane deflection principle. Figure 20a,b shows a detailed diagram and image of this sensing device. The device consists of a near-field communication (NFC) chip and coil antenna, which link to a sensor for pressure and temperature through a set of interconnections. The sensors are attached to a soft-printed circuit board (PCB) which acts as a base for these components. In Figure 20a,c platform is shown with a slim layer of PDMS encapsulation which preserves the electronic device and serves as a barrier to biofluids. According to the finite-element analysis (FEA), the tension in the copper layer is much lower than the yield strain (0.3%) when stretched by 8.5%, as demonstrated in Figure 20d. This elastic response—along with bending out of the plane, extending, and rotating—makes contact surfaces with the skin gentle and non-irritating, even at regions with a large degree of curvature, such as the rim of the heel. As displayed in Figure 20e–j, these devices are attached to diverse body positions that are prone to sacral ulcers, including the heel, malleolus, knee, elbow, scapulae, and sacrum.

**Figure 21:**
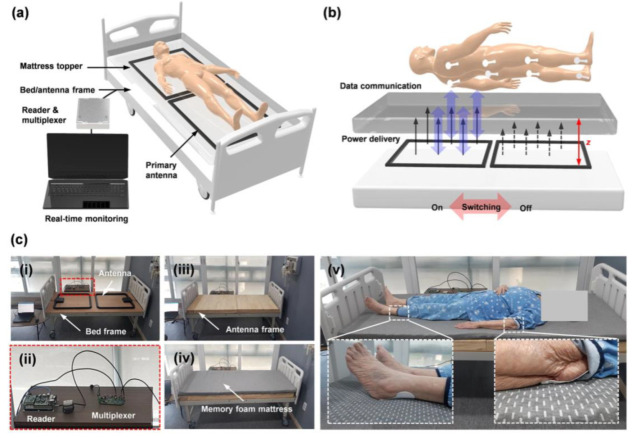
(**a**,**b**) Schematic diagram of the comprehensive system. (**c**) Images of primary antennas with multiplexing integrated into a hospital bed. Reprinted/adapted with permission from Ref. [6]. Copyright 2021, Springer Nature.

Using these sensing devices as a foundation, the researchers developed a comprehensive system that enables steady supervision of pressure and temperature at various points where the mattress contacts the skin of a patient lying in a medical bed [6]. This system is depicted in Figure 21a,b. Sensors placed at different positions provide real-time pressure and temperature readings using NFC protocols. The data is obtained through a rapid sequential readout system, which can record data from eight sensors per second. The system includes two primary antennas connected to a multiplexer and an NFC reader, all situated near the bed. In Figure 21c (i–iv), hardware installations of the system in a hospital ward are depicted, with the primary antennas attached to the bed frame and under the topper to capture data across the whole body of a typical subject (Figure 21c (v)). The researchers stated that clinical experiments with two hemiplegic patients and one tetraplegic patient proved the practicability, effectiveness, and sustainability of this technique in real-life medical settings.

### 4.3. Electronic Skin

The electronic skin is a new type of smart wearable sensing device which simulates the tactile perception of human skin, exhibiting similar thinness, softness, and stretchability. Furthermore, the development of flexible and stretchable sensing devices has enabled the electronic skin to detect a diversity of physiological signals, including biopotentials, temperature, and humidity, for applications in health monitoring [94,95] and human–machine interaction [96,97]. Recent developments in material and fabrication technologies have also enabled the integration of multiple sensors into a single electronic skin platform, providing multifunctionality and versatility for a range of applications.

**Figure 22:**
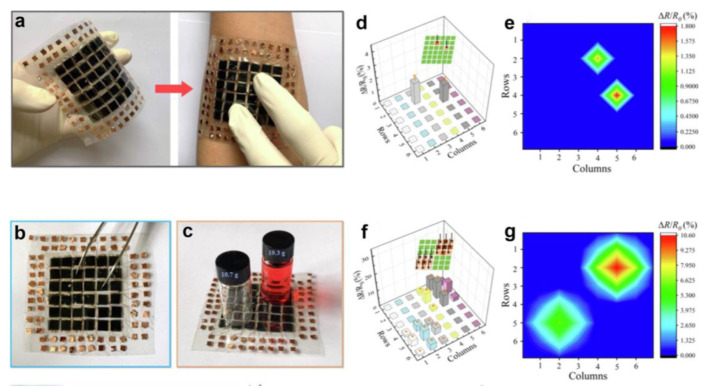
(**a**) Image of 6 × 6 array of interactive tactile sensors. (**b**) Image of point pressure on the device by a tweezer. (**c**) Image of plane pressure on the device by a bottle. (**d**–**e**) Distributions of point pressure (**d**,**e**) and plane pressure (**f**,**g**) on the device. Reprinted/adapted with permission from Ref. [14]. Copyright 2022, Elsevier.

Chen et al. proposed a micro-protruding structure MXene@PDMS piezoresistive pressure sensor using sandpaper as a template [14]. As can be seen in Figure 22a, they developed a 6 × 6 array of interactive tactile sensors, which can be stuck to the skin to map spatial variables. The resistance of each pixel was measured to generate tactile sensing images. The system accurately detected point pressure and showed corresponding resistance changes in local pixels (Figure 22b,d,e). When an empty bottle and a full bottle exerted plane pressure on it, the system generated 3D tactile sensing photographs that corresponded to the pressure distributions (Figure 22c,f,g). The images showed that the pressure by the fully filled bottle was higher than the empty one, owing to the emergence of more conductive pathways and the substantial decrease of the electrical resistance. Therefore, this sensor could be potentially useful in tactile sensing, electronic skin, and prosthetic devices.

**Figure 23:**
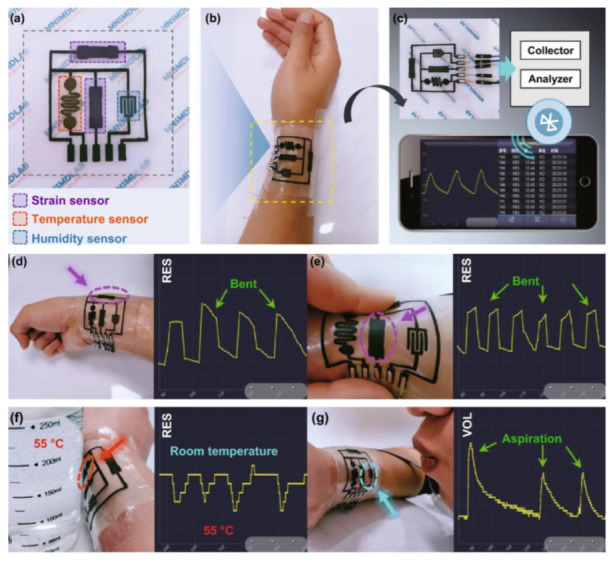
(**a**) Optical image of the device. (**b**) Image of the e-skin stuck to the wrist. (**c**) Schematic diagram of wireless monitoring system of the device. (**d**,**e**) Real-time response of wrist motion (d) and skin deformation (**e**) detected by the sensing unit and presented on a smartphone. (**f**,**g**) Changes in surrounding temperature (**f**) and humidity (**g**) measured by the temperature and humidity sensing unit and presented on a smartphone. Reprinted/adapted with permission from Ref. [98]. Copyright 2021, Springer Nature.

Lin et al. developed a simple and versatile method for fabricating self-healing e-skins using cellulose nanofiber/poly(vinyl alcohol) (CNF/PVA) composite material for the substrate and binder formation in the functional layers [98]. The screen-printing technique was used to fabricate a self-healable multifunctional e-skin with different functional units, including strain, temperature, and humidity units, exhibiting excellent performance and self-healing ability. Dousing the damaged section with water can self-heal both the functional layers and substrate in approximately 10 min. The e-skin shown in Figure 23a consisted of strain-sensitive, temperature-sensitive, and humidity-sensitive units arranged perpendicularly to each other. The functional units were deposited onto a CNF/PVA substrate with self-healing electrodes using screen-printing. The e-skin, connected to bluetooth transmission equipment and signal acquisition (Figure 23b,c), allowed for real-time wireless monitoring with test curves displayed on a mobile device. Wrist movement (Figure 23d) and skin deformation (Figure 23e) were monitored with the two strain sensors. Ambient temperature changes were monitored using the temperature sensor, which was tested by placing a cup of water at 55 °C near and away from the sensor (Figure 23f). The humidity sensor detected changes in ambient humidity, where exhaling air with high humidity resulted in a quick rise in humidity around the sensor (Figure 23g). In summary, this self-healing e-skin was integrated with traditional electronics for wireless transfer of data to neighboring smartphones, enabling real-time detecting of different external stimuli, which could be potentially useful in health monitoring and human–machine interaction.

### 4.4. Self-Powered Smart System 

With the rapid advancement of human–machine technology, there has been growing interest in developing integrated systems that include self-powered pressure sensors. These systems offer significant advantages in various applications. To achieve self-powered pressure sensors, an effective technique is to use nanogenerators based on nanocomposite materials. 

**Figure 24:**
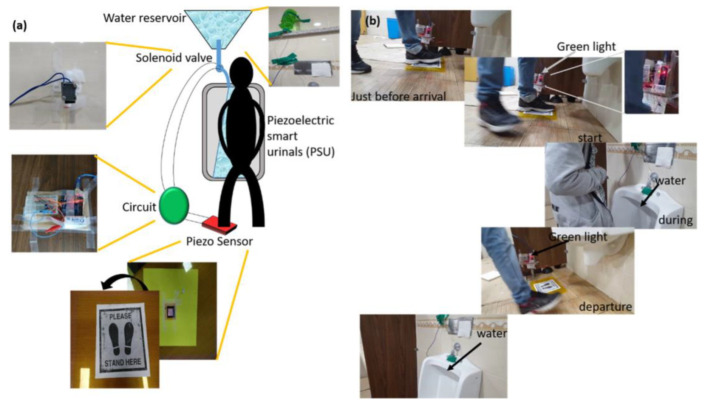
Self-powered smart urinal: (**a**) Illustration of the smart urinal system (**b**) The real-time image of the smart urinal system. Reprinted/adapted with permission from Ref. [99]. Copyright 2019, American Chemical Society.

Pandey et al. reported a type of nanogenerator based on nanocomposite materials, which was developed by simple grinding of n-ZnO:p-CuO heterojunctions (ZCH) [99]. This is a productive method for enhancing the piezoelectric capability of the ZCH/ PDMS composite. In conclusion, they developed a device that creates a piezoelectric-based smart urinal in response to the common problem of individuals neglecting to flush public urinals. Their solution is cost-effective and self-powered, as illustrated in Figure 24, and was developed using simple laboratory tools and an Arduino board with transistors. The Arduino board functions as a switch, detecting voltage signals from the device and opening the valve when triggered by the sensor. When a participant stands on the sensor, the board illuminates a green LED, signaling the valve to open and water to flow for 7.5 s. When the participant leaves, the sensor detects a signal, signaling the wall to flush for another 7.5 s, with a green LED illuminating the board to indicate completion.

## 5. Performance of Recently Reported Pressure Sensors with the Polymer Nanocomposites

Polymer composites for flexible pressure sensors mainly incorporate PS, PDMS, PVDF, PANI, PA, PEDOT:PSS, PET, PPy, PDA, Ecoflex, and hydrogel. Table 1 lists the newly developed pressure sensors based on these materials. Polymer composites for flexible pressure sensors have different advantages and disadvantages depending on the materials used. For example, PS and PDMS are soft and flexible, but they have low conductivity and stability. PVDF and PANI have high piezoelectric and conductive properties, but they are brittle and expensive. PA, PEDOT:PSS, PET, PPy, PDA and Ecoflex are relatively balanced in terms of flexibility, conductivity and stability, but they may have issues such as toxicity, degradation and hysteresis. Hydrogel has high water content, biocompatibility, and elasticity, but it has low mechanical strength, poor adhesion and swelling behavior. These pressure sensors have various applications in areas such as medical diagnosis, human–machine interaction, wearable devices and so on. The future development of these pressure sensors aims to improve their performance by optimizing the material composition, structure design, and fabrication methods.

## 6. Summary and Outlook

In this review, we offer a concise overview and discuss recent developments in flexible pressure sensors based on polymer nanocomposite materials. These sensors take on different forms, such as capacitive, piezoresistive, piezoelectric, triboelectric, and magnetoelastic sensors. The sensing principles and representative examples of these sensors are introduced and discussed. Numerous studies have demonstrated the feasibility of wearable sensing devices for health monitoring, electronic skin, and human physiology detection. As highlighted in this review, polymer-based pressure sensors have exhibited exceptional performance for different purposes.

In the future, polymer-based pressure sensors are expected to have more advantages over other sensors or sensing technologies, such as high sensitivity, low cost, simple fabrication, and biocompatibility. Some of the potential applications of these sensors include artificial organs, electronic skin, health monitoring, and environmental detection. To achieve these goals, some of the research directions that need to be explored are: (1) Developing novel polymer materials or composites with tunable electrical and mechanical properties. (2) Improving the sensitivity and selectivity to different types of pressure. (3) Integrating polymer-based pressure sensors with other types of sensing devices, such as temperature, humidity, or chemical sensors, to form multifunctional sensing devices. (4) Developing flexible and wearable power supply systems that can provide sufficient and stable energy for the sensors. (5) Optimizing the fabrication methods and scaling up the production of polymer-based pressure sensors for practical applications. (6) Enhancing reliability and durability under various environmental circumstances.

## Figures and Tables

**Table 1:** A summary of the performance parameters of flexible pressure sensors based on polymer composites.

Materials	Sensing Mechanism	Detection Limit	Response Time (ms)	Reliability (Cycles)	Sensitivity	Ref.
PS/PDMS	Capacitive	0.14 Pa	9	5000	44.5 kPa^−1^	[23]
MXene/PVDF-TrFE	Capacitive	1.5 Pa	150	10,000	0.51 kPa^−1^	[9]
Cu@Cu_2_O/GC	Capacitive	2.4 Pa	92	4000	90 kPa^−1^	[24]
glycerol/chitosan	Capacitive	-	180	1000	1.7 kPa^−1^	[25]
CNT/PDMS	Capacitive	-	60	5000	16.1% kPa^−1^	[26]
MWCNT/PDMS	Capacitive	5 Pa	43	1000	2.87 kPa^−1^	[49]
porous carbon/PDMS	Capacitive	4 Pa	60	11,000	1.1 kPa^−1^	[52]
CNT/PPy-PDA-PFDS	Capacitive	5 Pa	80	3000	147.4 kPa^−1^ (<20 kPa), 101.6 kPa^−1^ (20–50 kPa)	[97]
Cr/Cu/pyramid PDMS	Capacitive	0.655 Pa	81	1000	0.13674 kPa^−1^	[45]
Ti/Au/pyramid PDMS	Capacitive	-	346	-	0.16 kPa^−1^ (<1 kPa), 0.04 kPa^−1^ (1–2.5 kPa)	[46]
GO-PU nanofiber/ Ni-coated cotton yarn	Capacitive	0.001 N	50	1000	1.59 N^−1^ (<0.3N)	[94]
OVA/PAM hydrogel	Capacitive	4.4 Pa	18	1000	2.9 kPa^−1^	[100]
ZnSnO /silica fabric	Piezoresistive	-	350	500	0.12 kPa^−1^	[95]
MXene	Piezoresistive	4.4 Pa	130	10,000	151.4 kPa^−1^	[101]
MXene/PANI	Piezoresistive	-	106	10,000	690.91 kPa^−1^	[50]
CNT/PDMS	Piezoresistive	-	-	1500	0.1661 kPa^−1^ (0–18 kPa), 0.4574 kPa^−1^ (18–133kPa)	[58]
CNT/CB/CIP/silicone	Piezoresistive	1 Pa	88	40,000	0.136 kPa^−1^	[16]
MXene@PDMS	Piezoresistive	-	40	800	2.6 kPa^−1^	[14]
aPANF/GA	Piezoresistive	3 Pa	37	2600	28.62 kPa^−1^	[30]
CNT/PDMS	Piezoresistive	0.2 Pa	40	-	15.1 kPa^−1^	[102]
MXene/rGO aerogel	Piezoresistive	6 Pa	232	6000	609 kPa^−1^	[27]
PEDOT:PSS/PDMS	Piezoresistive	7.14 Pa	0.2	10,000	642.5 kPa^−1^	[103]
PEDOT:PSS/PPy	Piezoresistive	-	0.36	-	0.58 kPa^−1^	[104]
CB/TPU	Piezoresistive	10 Pa	18	10,000	5.54 kPa^−1^	[105]
AuNWs/PDMS	Piezoresistive	-	20	10,000	0.03 kPa^−1^	[106]
PPy/PDMS	Piezoresistive	1 Pa	20	1000	19.32 kPa^−1^	[107]
SA/PAA/ Al^3+^/Mxene hydrogel	Piezoresistive	-	210	400	0.075 kPa^−1^	[108]
HLP-hydrogel	Piezoresistive	-	152	300	0.131 kPa^−1^ (0–15 kPa) 0.051 kPa^−1^ (15–50 kPa)	[109]
LBG/PVA/CNTs hydrogels	Piezoresistive	-	356	1000	20.5 kPa^−1^ (0–1 kPa)	[110]
GaN NWs	Piezoelectric	-	55	-	14.25 V·kPa^−1^	[32]
(K,Na)NbO3	Piezoelectric	20 g	-	10,000	0.20 V·N^−1^	[77]
ZnO/PDMS	Piezoelectric	-	-	-	1.7 V·N^−1^	[99]
ZnO-PDMS	Piezoelectric	-	15	3000	70.8 mV·N^−1^	[33]
PVDF/rGO/BT	Piezoelectric	-	-	-	7.34 mV·kPa^−1^	[71]
PDA@BTO/PVDF	Piezoelectric	-	-	7400	3.95 V·N^−1^	[70]
ZnO nanofibers/PVDF	Piezoelectric	1.8 Pa	53	5000	3.12 mV·kPa^−1^	[111]
FEP/Ecoflex	Piezoelectric	125 Pa	18.6	3600	32.6 nA·kPa^−1^	[112]
ZnO/conductive fabric	Piezoelectric	-	-	-	0.08 mV·kPa^−1^	[113]
MXene/PDMS	Triboelectric	-	30	6000	651.37 mV·N^−1^ (0.15–2.50N), 467.74 mV·N^−1^ (2.50–5.15N)	[51]
FEP/PA	Triboelectric	5 mg	30	20,000	10.29 nA·kPa^−1^	[79]
PDMS-microsphere	Triboelectric	-	-	5000	150 mV·Pa^−1^	[114]
graphite/PDMS	Triboelectric	-	-	15,000	7.3 V·Pa^−1^	[37]
PAM/BTO	Triboelectric	-	70	4500	7.91 V·N^−1^ (<1N)	[31]
PET/Nylon/Stainless steel	Triboelectric	100 Pa	-	16,000	1.1 V·kPa^−1^ (100 Pa-11 kPa) 0.063 V·kPa^−1^ (11 kPa-400 kPa)	[96]
PTFE/PA/PVDF	Piezo/ triboelectirc	-	-	15,000	1.30298 V·N^−1^ (0.4–1.6N), 6.2249 V·N^−1^ (1.6–2.8N)	[83]
PZT/PDMS	Piezo/ triboelectirc	-	45	10,000	18.96 V·kPa^−1^ or 230 nA·kPa^−1^	[10]
Ecoflex/NdFeB/PA	Magnetoelastic	-	15	5000	0.27 mV·kPa^−1^ (<2 Hz)	[43]
Ecoflex/NdFeB/PA	Magnetoelastic	0.05 kPa	5	10,000	-	[41]

PS: polystyrene; PDMS: polydimethylsiloxane; PVDF-TrFE: poly(vinylene fluoride-trifluoroethylene); GC: graphitic carbon; CNT: carbon nanotubes; MWCNT: multiwall carbon nanotubes; OVA: ovalbumin; PAM: polyacrylamide; PANI: polyaniline; CB: carbon blacks; CIP: carbonyl iron powder; aPANF/GA: a novel nanofiber reinforced graphene aerogel; rGO: reduced Graphene Oxide; NWs: nanowires; BT: barium-titanium oxide; PDA: polydopamine; PVDF: polyvinylidene fluoride BTO: barium titanate; PEDOT:PSS: poly(3,4-ethylenedioxythiophene) polystyrene sulfonate; PPy-PDA-PFDS: polypyrrole-polydopamine-perfluorodecyltrlethoxysilane; GO-PU nanofiber: graphene-doped polyurethane nanofiber; TPU: thermoplastic urethane elastomer; SA: Sodium alginate; PAA: polyacrylic acid; HLP-hydrogel: hybrid latex particle cross-link hydrogel; FEP: fluorinated ethylene propylene; PA: polyamide; PTFE: polytetrafluoroethylene; PZT: lead zirconate titanate.
